# On the Cause of Inflammatory Diseases

**Published:** 1810-09

**Authors:** 


### *To the Editors of the Medical and Physical Journal.*

GENTLEMEN,

I Believe it to be generally acknowledged by medical practitioners, that inflammatory diseases have been more prevalent within these few years than at any former period. To ascertain the cause of this is a task of a difficult nature, and I have not the vanity to suppose that any thing I can advance upon this part of the subject, will tend in the least to lessen that difficulty; it is generally understood that a plethoric habit of body is one of the predisposing causes to those diseases, but we find the spare delicate frames of females almost as much subject to the influence of inflammatory diseases as the more stout and athletic of the male sex. Violent exercise is stated to be an exciting cause; but the indolent and inactive are no less liable to inflammatory attacks. The intemperate use of spirituous liquors is assigned as a frequent cause, and so it may be, in some instances, but yet the temperate are not exempt. I have had an opportunity of witnessing two or three instances of this kind *lately*, in individuals who had invariably accustomed themselves to the most systematic and regular mode of living, labouring under violent inflammatory affections. The state of the atmosphere is generally resorted to as a dernier resource, when no other cause can be assigned, and perhaps this may be the most rational cause of all. The alterations of temperature observable in this variable climate, may certainly have great influence in deranging the actions of the human frame, and great care ought, at all times, to be observed to prevent the *sudden application* of either cold or heat. To be acquainted with the causes of disease, though gratifying to the physiological enquirer, is not absolutely of so much consequence as to be in possession of remedies capable of successfully combating those diseases. Till within late years, the internal and external exhibition of mercury was almost entirely tirely restricted to the prevention and cure of syphilis; but as the nature of the disease became more investigated, and the action of mercury on the system *better* understood, practitioners of discernment have been disposed to try its effects in other complaints, and certainly with success.

It is one of the late Mr. J. Hunter's physical axioms, that no two actions can exist in the same system, at the same time. If this is fact, and I am disposed to think it is from observation, with two or three exceptions, is it not rational to suppose, that the action of mercury on the system, opens a wide field for improvement in the treatment of diseases? I have had the pleasure to observe its *marked utility* both in acute rheumatism and acute hepatitis; in both diseases, the same plan of treatment was adopted; in order to lower excessive action, bleeding was resorted to in the first instance, the extent to which it was carried, regulated by the *rise*, *strength*, and hardness of the pulse; the bowels were cleared. Mercury was then exhibited in appropriate doses, augmenting the dose till a *slight* ptyalism was produced; it became necessary in general to join an opiate with the calomel, in order to obviate its purgative effects on the bowels, and at the same time, to allay pain. *Great* relief was experienced while under the influence of the mercury before it affected the mouth; but when that became affected, a striking remission of pain and every other symptom was felt; the disease imperceptibly vanished, leaving the patient in a state of convalescence. Tonics were then resorted to, to restore the tone and strength of the system; and a perfect recovery took place. To have inserted the minutes of these cases would have taken up much more room than is consistent with the accustomed plan of your work. The grand query to which I would solicit attention is, whether the exhibition of mercury may not be attended with advantage in every inflammatory disease, upon this principle, that by affecting the system with it, and inducing a new action, you supersede the existing morbid one, and if not entirely destroy it, at least, in a great degree, mitigate its effects? One caution it may be necessary to make, that its exhibition ought never to be entrusted to any but scientific enlightened men, who narrowly watch its effects, and are aware of the principle upon which it acts. Should these remarks tend in the *least* to excite an attention in the minds of medical men, to the subject in question, the end of the writer will be fully answered.

I am, &c.

*July* 25, 1810. VERAX

